# Tin nanoparticles as an effective conductive additive in silicon anodes

**DOI:** 10.1038/srep30952

**Published:** 2016-08-03

**Authors:** L. Zhong, C. Beaudette, J. Guo, K. Bozhilov, L. Mangolini

**Affiliations:** 1 Materials Science and Engineering Program, UC Riverside, Riverside CA, USA; 2 Mechanical Engineering Department, UC Riverside, Riverside CA, USA; 3 Chemical and Environmental Engineering Department, UC Riverside, Riverside CA, USA; 4 Central Facility for Advanced Microscopy and Microanalysis, UC Riverside, Riverside CA, USA

## Abstract

We have found that the addition of tin nanoparticles to a silicon-based anode provides dramatic improvements in performance in terms of both charge capacity and cycling stability. Using a simple procedure and off-the-shelf additives and precursors, we developed a structure in which the tin nanoparticles are segregated at the interface between the silicon-containing active layer and the solid electrolyte interface. Even a minor addition of tin, as small as ∼2% by weight, results in a significant decrease in the anode resistance, as confirmed by electrochemical impedance spectroscopy. This leads to a decrease in charge transfer resistance, which prevents the formation of electrically inactive “dead spots” in the anode structure and enables the effective participation of silicon in the lithiation reaction.

Silicon has been intensively investigated as next generation anode material for lithium ion batteries because of its high lithiation capacity and because it is generally considered an earth-abundant, non-toxic material^1^,^2^,^3^,^4^,^5^,^6^. Despite these advantageous properties, several factors have hampered its successful integration in real-life batteries. Its structural instability upon lithiation, its low electrical conductivity and its tendency to form a mechanically unstable solid electrolyte interphase (SEI)^7^ lead to rapid capacity fading. Several approaches to mitigate these issues are discussed in the large and growing literature on this topic. The use of silicon nanoparticles can prevent the pulverization and loss of the active material^8^, although it comes at the price of an increase in specific surface area which negatively affects the first cycle coulombic efficiency. Strategies have been proposed to effectively reduce the active material surface area while maintaining its nanostructure^9^,^10^. Additives to the electrolyte such as fluoroethylene or vinylene carbonate (FEC, VC) promote the formation of a thinner and more stable SEI^11^,^12^,^13^. Several studies have focused on the development of novel binders which can tolerate the large volume changes associated with the lithiation of silicon^14^,^15^,^16^. The issue of the poor electrical conductivity of silicon has also been addressed mainly by combining it with various forms of carbon^10^,^17^,^18^,^19^,^20^. Promising results have been attained by over-coating silicon nanostructures with carbon layers either via chemical vapor deposition^20^,^21^ or by thermally decomposing a polymer additive^10^. It is well-known that the conductivity of silicon-based active layers can be improved by increasing the weight fraction of conductive carbon-based additives such as nanotubes or carbon black, but this approach inevitably “dilutes” the active layer, i.e. decreases its average gravimetric and volumetric capacity^22^,^23^. This last consideration motivates this study: tin has significantly higher electrical conductivity compared to silicon (10^−7^ Ω·m versus 2 × 10^3^ Ω·m). It has high volumetric density and a maximum theoretical capacity upon lithiation (994 mAh/g) that also exceeds that of graphite^24^. The original idea motivating this study was to use tin nanoparticles as an effective conductive additive that does not “dilute” the active material, since tin has both high conductivity and good capacity for lithiation. We have found that the benefits of using tin as an additive go beyond those outlined above. The addition of even minor amounts (as low as 2.2% by weight) of tin to a silicon-containing anode can lead to significant improvements in anode performance. Our data suggest that by uniformly dispersing small tin nanoparticles in a mesoporous silicon network it is possible to significantly decrease the electrical resistance of the structure, facilitating the lithiation of silicon and achieving >80% first cycle coulombic efficiency, followed by <20% capacity decay over 100 cycles at a 0.1 C cycling rate. The same mesoporous, silicon-based structure would rapidly fail upon cycling without the addition of tin.

## Methods

Silicon nanoparticles (<100 nm, SiNPs), tin dichloride (SnCl_2_·2H_2_O, 98% purity), polyvinylpyrrolidone (PVP, molar weight 40,000) were purchased from Sigma-Aldrich, and used as received. Carbon black Super P (CB) was purchased from Alfa Aesar. SiNPs, SnCl_2_·2H_2_O, CB and PVP were weighted and mixed in ethanol by probe sonication. Typically, 100 mg of SiNPs are mixed with 19 mg of SnCl_2_·2H_2_O, 10 mg of carbon black and 360 mg of PVP in ethanol and probe sonicated for 10 mins. The ethanol-based slurry is then coated onto copper foil (from MTI Corp.) via Mayer rod and let evaporate in air. The film is then annealed at 700 °C under argon for 15 minutes. After cooling, the samples are extracted from the annealing furnace, and ½ inch diameter circles are punched out for electrode preparation. The typical weight loading for the samples discussed in this manuscript is 0.4 mg/cm^2^. The elements weight ratio is determined by SEM-EDS.

The CR2032 typed coin cells are assembled in an argon-filled glove-box with a polymer separator (MTI) and lithium metal foil (Alfa Aesar) as counter electrode. A 1 M solution of lithium hexafluorophosphate (LiPF_6_) in 1:1 v/v ethylene carbonate/diethyl carbonate (Sigma-Aldrich) is used as electrolyte. Fluoroethylene carbonate (FEC) (Solvay S.A., Belgium) is added to the electrolyte at 10% volume fraction. Coin cells are cycled between 0.01 V and 1.5 V using a battery tester from Arbin Instruments. Cyclic voltammetry (CV) is performed at a sweeping rate of 0.1 mV/s using model VMP3 from BioLogic Science. Electrochemical impedance spectroscopy (EIS) is performed on a Gamry potentiostat with frequency range from 10 KHz to 0.1 mHz.

The structures under investigation have been characterized using standard techniques such as x-ray diffraction (XRD), scanning electron microscopy (SEM) and transmission electron microscopy (TEM). SEM is performed on a Nova NanoSEM 450 with elemental analysis capability (EDS). TEM samples are prepared by scratching some of the active material onto a lacey carbon grid. TEM analysis is performed on a FEI Titan Themis 300. XRD is performed on a PANalytical EMPYREAN instrument with a CuKα source.

## Results and Discussion

**Figure 1:**
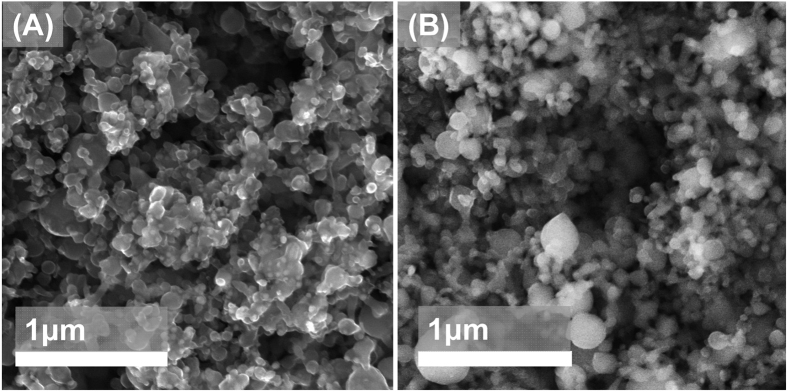
(**A**) Top-down SEM of the active layer after coating and annealing, without the addition of the tin precursor to the slurry. (**B**) Same as (**A**), but with the addition of the tin precursor.

Figure 1A,B show top-down SEM images of two electrodes prepared with and without the addition of SnCl_2_·2H_2_O to the formulation, after annealing at 700 °C in argon and before cycling. The anodes consist of mesoporous layer in which the silicon nanoparticles are clearly distinguishable. EDS analysis on the electrode without tin reveals that the silicon weight fraction is 71%, the oxygen weight fraction 9% and the carbon weight fraction is 20%. The carbon contribution results from both the presence of carbon black particles and from the thermal decomposition of the polymer precursor, PVP, which results in the formation of an amorphous carbon layer surrounding the nanoparticles, as extensively discussed in our previous work^10^. The addition of SnCl_2_·2H_2_O to the formulation leads to the inclusion of tin into the structure, although we have found that a partial loss of tin is inevitable during the annealing process. This is expected given the high vapor pressure of tin. For instance, when targeting a 10:1 Si:Sn weight ratio (corresponding to a formulation containing 100 mg of SiNPs and 19 mg of SnCl_2_·2H_2_O) we measure a 2.2% weight fraction of tin by SEM-EDS based on the total mass of the electrode. A comparison of Fig. 1A,B confirms that the morphology of the coating, after annealing, is not affected by the presence of tin.

**Figure 2:**
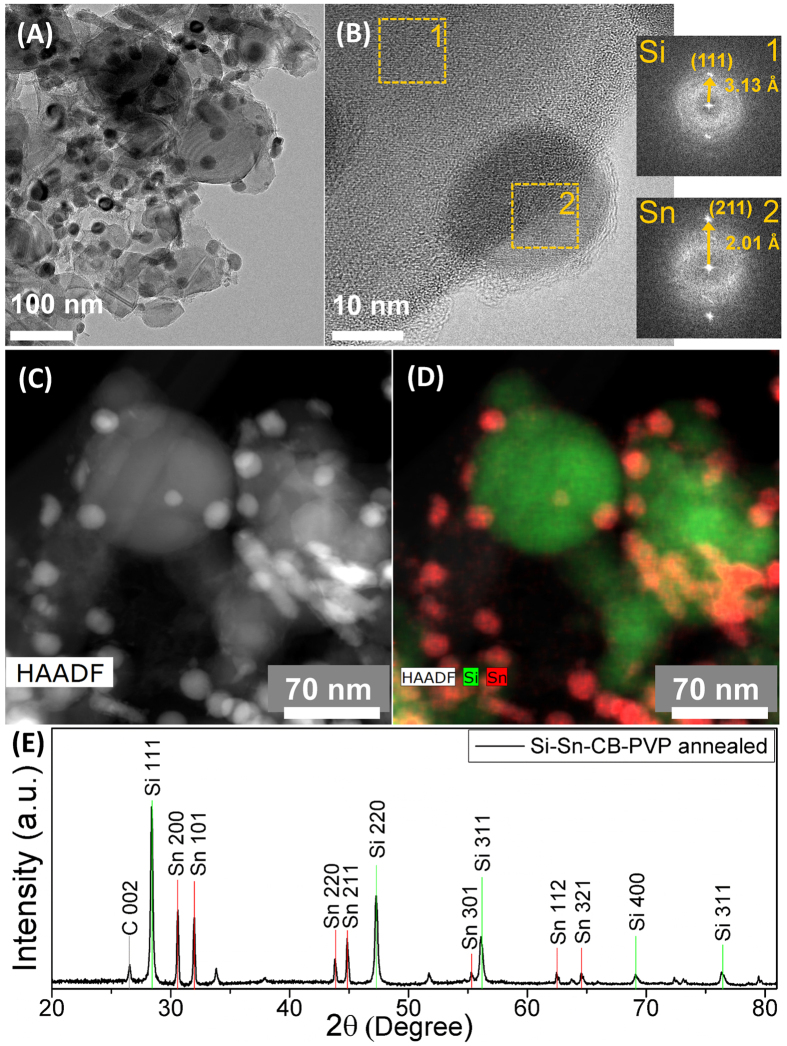
(**A**) TEM bright field image of the silicon-tin nanocomposite, prepared by scratching some of the film from the copper foil after annealing. (**B**) Higher magnification TEM image for the same sample shown in (**A**). The FFT patterns for regions 1 and 2 are also shown. (**C**) High angle annular dark field image for the sample shown in (**A**), confirming the compositional variation between the large particles and the smaller one decorating the surface of the larger particles. (**D**) Elemental mapping corresponding to (**C**), confirming that the larger particles are silicon while the smaller ones are tin. (**E**) XRD pattern for the silicon-tin nanocomposite, confirming the presence of both the silicon and the tin crystalline phases.

Figure 2 summarizes the results from the structural characterization of the anode material. TEM analysis (Fig. 2A) indicates that the layer is composed of agglomerated nanoparticles, with larger particles in the 100 nm size range whose surface is surrounded by smaller particles in the 10–20 nm size range. Higher resolution analysis (Fig. 2B) suggests that the larger particles are silicon while the smaller one are tin, as confirmed by the FFT of the higher resolution image for two distinct regions of interests. A lattice spacing of 3.13 Å is measured for the larger particles, corresponding to the interplanar distance between the (111) planes of silicon, while a spacing of 2.01 Å is measured for the smaller particle, corresponding to the (211) interplanar distance of tin. The high-angle annular dark field image (HAADF) in Fig. 2C shows significant contrast between the larger and the smaller particles, and the corresponding elemental mapping shown in Fig. 2D leads us to conclusively confirm that the anode is composed of larger SiNPs surrounded by smaller SnNPs. The SiNPs and the SnNPs are coated with a thin carbon layer. This is a consequence of the thermal decomposition of PVP during the annealing step. In our previous studies we have observed a 90–95% weight loss for PVP during annealing under argon, with the residue being composed of amorphous carbon^10^.

The carbon layer that surrounds both silicon and tin particles is critical at maintaining the structural integrity of the film. Figure S1 in the Supplementary Information file shows a silicon nanoparticle surrounded by a 5–10 nm thick amorphous carbon shell, the result of the decomposition of the polymer precursor. Figure S2 in the Supplementary Information file shows a tin nanoparticle surrounded by a few nm thick carbon layer. Figure S3 in the Supplementary Information file shows smaller tin nanoparticles surrounding a larger silicon particle. A thin carbon shell can be seen surrounding the particles. Figure S4 focuses on the interface between the silicon and the tin nanocrystals. A thin amorphous layer, likely amorphous carbon, separates the two particles. In Fig. S5 we show a high-angle annular dark field image (HAADF) similar to that shown in Fig. 2C. The corresponding elemental map for silicon and tin is also shown, demonstrating that small tin particles surround the silicon particles. The elemental map for carbon is also shown in Fig. S5, confirming that the carbon signal overlaps with that from both silicon and tin and suggesting that a carbon layer surrounds the structure.

The composite is also examined via XRD. The diffraction spectrum is shown in Fig. 2E. Peaks due to metal Sn nanocrystals (200 at 30.5°, 101 at 32°, 220 at 44°, 211 at 45° and 301 at 56°) and due to Si nanocrystals (111 at 28°, 220 at 47.3°, 311 at 56.1°, 400 at 68.9°) can be easily distinguished. We could resolve a contribution from crystalline carbon at 26.5°, the result of the presence of carbon black in the anode. The peak assignment is based on the ICSD database (Inorganic crystal structure database FIZ Karlsruhe). The XRD analysis is consistent with TEM results.

While a detailed explanation of the formation of SnNPs via thermal decomposition of SnCl_2_·2H_2_O in presence of a polymer are left for a future study, we point out that a similar approach has already been reported for the synthesis of SnNPs dispersed in a carbon matrix^25^,^26^.

**Figure 3:**
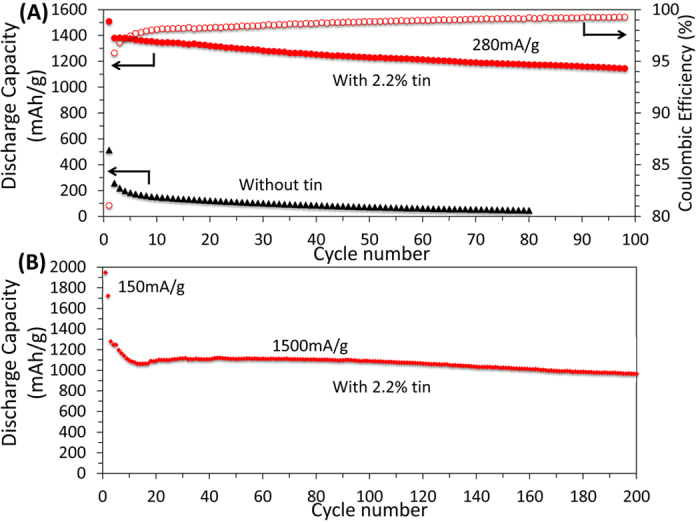
(**A**) Galvanostatic discharge capacity performance of the anode containing 2.2% of tin, by weight (red) and for the anode without tin (black) cycled at 0.1 C rate. The open red circles correspond to the coulombic efficiency (left axis) for the anode with tin. (**B**) Galvanostatic discharge capacity performance of anode containing 2.2% by weight of tin, cycled at 1 C rate. The specific capacities are calculated based on the total weight.

The electrochemical performance for the anode composed of a silicon-tin nanocomposite (with 2.2% of tin by weight) is shown in Fig. 3A. The performance of the control structure without any tin is shown in the same plot. The anodes were cycled at 0.1 C (∼280 mA/g). The tin-containing electrode (red dots) shows superior performance compared to the electrode without tin (black dots) with respect of both energy storage capacity and cycle stability. All the specific capacities are calculated based on the total weight of the electrode materials. The tin-containing anode has a first cycle discharge capacity and columbic efficiency of 1500 mAh/g and 81% respectively, and maintains a capacity exceeding 1100 mAh/g after 100 cycles, corresponding to a >75% capacity retention with respect of the first cycle. In contrast, the anode without tin has a capacity lower than 200 mAh/g after just 4 cycles. The coulombic efficiency for electrode with 2.2% tin reached to 99.3% at 100 cycles.

Figure 3B shows the performance of a tin-containing anode at a higher cycling rate. The first two cycles are completed at 150 mA/g, which is commonly done to ensure that the whole active layer is lithiated. The following 200 cycles are performed at a 1.5 A/g rate. The anode has a capacity of 970 mAh/g even after 200 cycles, demonstrating good stability even at higher charge-discharge rate. Based on the data presented in Fig. 3, we can conclude that the addition of even a minor amount of tin of the anode structure leads to dramatic improvements with respect of both capacity and stability.

**Figure 4:**
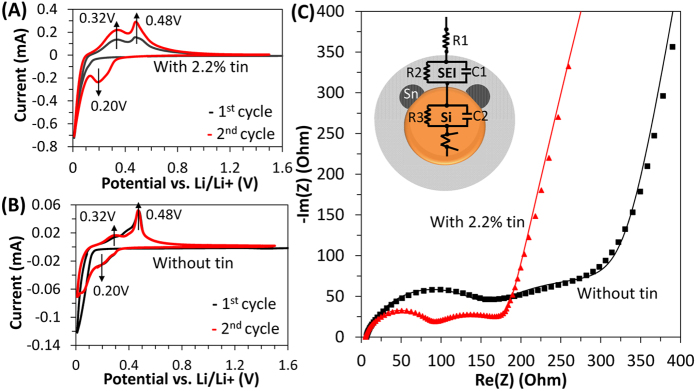
(**A,B**) Cyclic voltammetry for the anodes with 2.2% tin by weight and without tin respectively. (**C**) EIS curves after one cycle for the anode without tin and for the anode with 2.2% tin. Both experimental and fitted model data are shown. In the inset we show a schematic of the model used to interpret the EIS data.

In order to elucidate the mechanism leading to this improvement, we have performed additional characterization focusing on the electrochemical behavior of the structures under consideration. In Fig. 4A,B we show the cyclic voltammetry measurements for the first and second cycles for the anodes with and without tin addition respectively. The weight fraction of tin is 2.2%, the same as for the anodes discussed previously. The measurements were performed at a sweep rate of 0.05 mV/second. For the anode with tin (Fig. 4A), we can observe a clear lithiation peak at 0.20 V and delithiation peaks at 0.32 V and 0.48 V, corresponding to the theoretical potentials for silicon^3^. The fact that the lithiation peak is not present in the first cycle is not surprising, since it is known that silicon-based anodes go through a structural transformation during the first cycle which leads to the appearance of the 0.20 V lithiation peak at the second cycle^3^. As a comparison, the anode without tin (Fig. 4B) also shows a lithiation peak at 0.20 V for the second cycle, although this peak is weaker compared to the case of the tin-containing anode. A second broad feature around 0.08 V is observed, also corresponding to lithiation of silicon. The delithiation peaks at 0.32 V and 0.48 V are still present, although the 0.48 V appears to be more prominent. Both anodes show lithiation and delithiation peaks at the theoretically predicted potentials. We interpret these observations in terms of either a higher over-potential requirement for the lithiation-delithiation reaction, or of a less uniform potential distribution in the mesoporous structure for the anode without tin. A combination of these two phenomena is likely. Lithiation peaks for Sn are at 0.37 V and 0.62 V, while oxidation peaks are at 0.67 V and 0.8 V according to literature^24^. The tin-related lithiation and delithiation peaks are not observed in the scan shown in Fig. 4A, likely because of the small weight fraction of tin in the structure. In Fig. S6 of the Supplementary Information file we show the CV scan for an anode containing a 9.6% weight fraction of tin, confirming that tin participates in the lithiation reaction.

In addition to the cyclic voltammetry data, we have performed electrochemical impedance spectroscopy (EIS) on anodes with and without tin. The Nyquist diagram for these structures is shown in Fig. 4C. We have fit the experimental data using a model which includes a series of three impedances and a Warburg element. Such models have been utilized by several groups to fit the EIS data^18^,^27^. The inset of Fig. 4C shows a schematic which describes our physical interpretation of the three-impedance model. R1 represents the resistance due to the electrolyte. The contribution from the second resistance (R2) appears in the high frequency semicircle, and accounts for the resistance between the electrolyte and the active materials. Its value is determined by the presence of the solid electrolyte interphase (SEI) layer surrounding the active material. The mid-frequency semicircle represents the charge-transfer resistance (R3) in the active layer (composed of SiNPs, SnNPs, carbon black and amorphous carbon). A Warburg element, describing lithium ion diffusion in the active layer, is used to fit the low frequency region. The results of the fitting procedure are summarized in Table 1. The value of R1 is in the 5–10 Ohm range for the structures under consideration. Similar values have been reported in the literature for this component^27^. The value of the second resistance, R2, decreases when tin is added to the anode. It is 140.2 Ohm for the silicon-only electrode and 100.2 Ohm for a 2.2% weight fraction of tin, corresponding to roughly a 30% decrease in resistance. The third resistance, R3, is much larger for the electrode without tin (177.8 Ohm) compared to the electrode with tin (82.7 Ohm), corresponding to >50% decrease in resistance. All anodes show a very similar slope in the low frequency region, suggesting similar ion diffusion kinetics^28^.

This data conclusively confirms that the addition of even minor amounts of tin to the film leads to a significant reduction in R2, implying that the SEI-related resistance is lowered by the addition of tin. TEM and STEM analyses indicate that tin nanoparticles surround the surface of the silicon particles, i.e. they are in direct contact with the the SEI layer. This configuration, which is achieved by the simple addition of a soluble tin precursor such as tin dichloride to the silicon-containing slurry, reduces the SEI-related resistance. In Fig. S7 of the Supplementary Information file we show the same EIS analysis for the anode containing a 9.6% weight fraction of tin. An increase in tin content leads to a further decrease in the value of R2 (from 100.2 to 78.09 Ohm, roughly a 20% decrease). This is consistent with increased weight loading of tin which further reduces the resistance introduced by the SEI layer. The value of R3 increases slightly (from 82.7 to 93.9 Ohm, a 13% increase), which may be due to small differences in actual weight loading between these two anode. We have kept the coating parameters (slurry concentration and wet coating thickness) as close as possible between samples, but variations in the wet coating thickness can be as high as 20% when coating via hand drawdown^29^. Nevertheless, the addition of even 2.2% by weight (roughly 0.7% by volume) of a highly conductive element such as tin contributes to the decrease in the overall resistance. To explain this observation, we should point out that the first cycle capacity for the silicon-only electrode is lower than for the silicon-tin nanocomposite case (∼500 mAh/g vs. ∼1500 mAh/g). In addition, XRD analysis on the active layer after the first charge-discharge cycle shows that the silicon peaks are still detectable for the active layer without tin (see Fig. S8 in the supporting information file), while they are undetectable for the tin-containing case. These data suggest that for the silicon-only case the active layer is not fully lithiated after the first cycle. This explains the higher value of R3, since the EIS measurements are performed after the first lithiation process (the half-cell is discharged to 0.01 V and rested for 60 mins before the EIS measurement). For the silicon-only case, a fraction of the active layer is composed of non-lithiated silicon which has significantly higher resistance than the lithiated phase.

**Figure 5:**
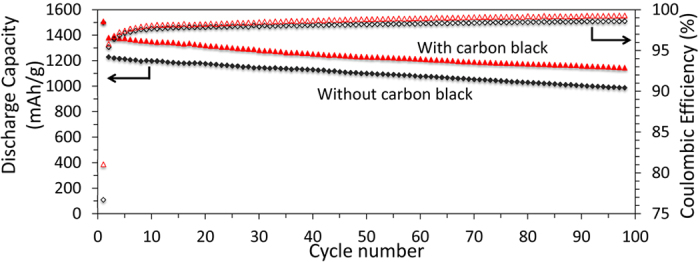
Cycle performance of anodes produced with (red) and without (black) carbon black added to the structure. All discharge capacities were calculated based on total electrode.

The combination of the cyclic voltammetry and the impedance spectroscopy measurements lead us to the following conclusions: (a) tin participates in the lithiation reaction and contributes to the total energy storage capacity of the anode, although its contribution cannot explain the improvement in capacity shown in Fig. 3A because of the very small fraction of tin present in these anodes; (b) even minor addition of tin nanoparticles lead to a dramatic decrease in the overall resistance of the anode, in particular by reducing the potential loss across the SEI layer, resulting in vast improvements in both capacity and stability. This implies that one of the main mechanisms causing the poor performance of the silicon mesoporous layer is the fact that the structure does not fully participate in the lithiation reaction. This is a consequence of a non-uniform potential distribution which leads to the formation of “electrical dead spots”, i.e. portions of the films that do not reach the lithiation potential and are therefore inactive. The addition of tin to the electrode offers a simple solution to this problem, since it effectively enables the lithiation-delithiation of the active material. The fact that the anode still shows capacity fading is not unexpected because this simple structure is not designed to withstand the strain accumulated over several cycles. The effectiveness of the tin addition is confirmed by the result of an additional experiment, in which we have compared the performance of anodes produced with and without carbon black added to the formulation. The tin concentration is around 2% by weight for both anodes. The galvanostatic cycling performance for the two structures is shown in Fig. 5.

The first cycle capacity is practically identical for both electrodes and equal to 1500 mAh/g. The first cycle coulombic efficiencies are 81% and 76% for the anode with and without carbon black respectively. After 100 cycles at 0.1 C, the capacities are 1100 mAh/g and 920 mAh/g for the anode with and without carbon black respectively. The corresponding coulombic efficiencies are 99.3% and 98.6%. We conclude that the addition of carbon black is beneficial, but if we compare these results to those shown in Fig. 2A, in which the anode with carbon black but without tin shows rapid capacity fading, we can conclude that the addition of tin is the crucial factor that enables the anode functionality. This last experiment highlights the importance of achieving a uniform mixture between the active component (SiNPs) and the conductive additive. The approach described in our study, i.e. the addition of a soluble tin precursor to the silicon-containing slurry, is simple and effective at attaining this goal.

## Conclusion

We have described the fabrication and testing of silicon-tin nanocomposite anodes for lithium ion batteries. We have used commercial silicon particles and off-the-shelf additive such as tin dichloride and PVP to realize anodes that show good performance in both capacity and stability. These structures show a dramatic improvement compared to those prepared without tin. EIS measurements suggest that these composites have overall lower active layer resistance compared to the silicon-only case. This avoids the formation of electrical “dead spots”, and enables the full utilization of the active material. These results have been obtained using a simple, mesoporous silicon film as control structure, therefore the stability of our best device is still not compatible with the >1000 cycles lifetime required in real-life batteries. Still, the effectiveness of this simple, low-cost approach suggests that if used in combination with more advanced structures, it may be provide the critical improvement necessary to finally realize a silicon-based next-generation anode.

## Additional Information

**How to cite this article**: Zhong, L. *et al*. Tin nanoparticles as an effective conductive additive in silicon anodes. *Sci. Rep*. **6**, 30952; doi: 10.1038/srep30952 (2016).

## Supplementary Material

Supplementary Information

## Figures and Tables

**Table 1:** Summary of the impedance spectroscopy data analysis.

	R1 (Ω)	R2 (Ω)	R3 (Ω)
Without tin	6.2	140.2	177.8
2.2 wt% tin	10.9	100.2	82.7
