# Supplementary material for: Tin nanoparticles as an effective conductive additive in silicon anodes
Source: Sci Rep. 2016 Aug 3;6:30952. doi: 10.1038/srep30952 (PMC4971508; doi:10.1038/srep30952)
Supplement: Supplementary Information [file srep30952-s1.doc]

**SUPPLEMENTARY INFORMATION**

**Tin nanoparticles as an effective conductive additive in silicon anodes**

L. Zhong^a^, C. Beaudette^b^, J. Guo^a,c^, K. Bozhilov^a,d^, L. Mangolini^a,b^

a) Materials Science and Engineering Program, UC Riverside, Riverside CA, USA

b) Mechanical Engineering Department, UC Riverside, Riverside CA, USA

c) Chemical and Environmental Engineering Department, UC Riverside, Riverside CA, USA

d) Central Facility for Advanced Microscopy and Microanalysis, UC Riverside, Riverside CA, USA


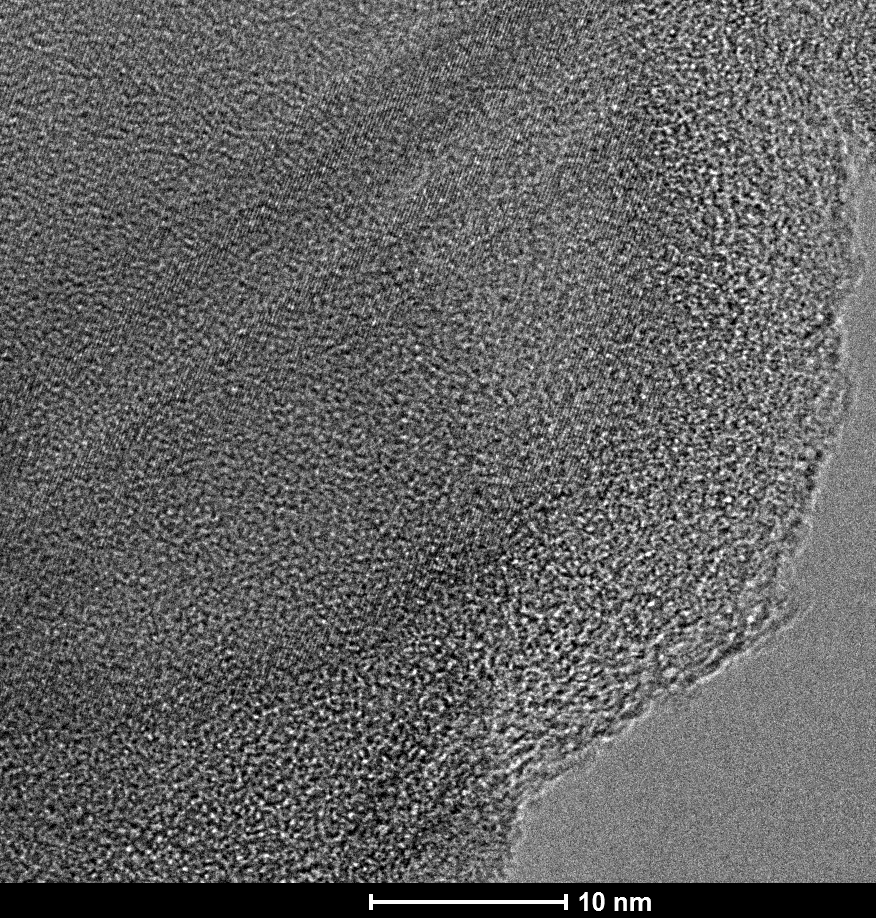


Figure S1. TEM of a silicon nanoparticle surrounded by a thick amorphous carbon layer resulting from the decomposition of the polymer precursor.


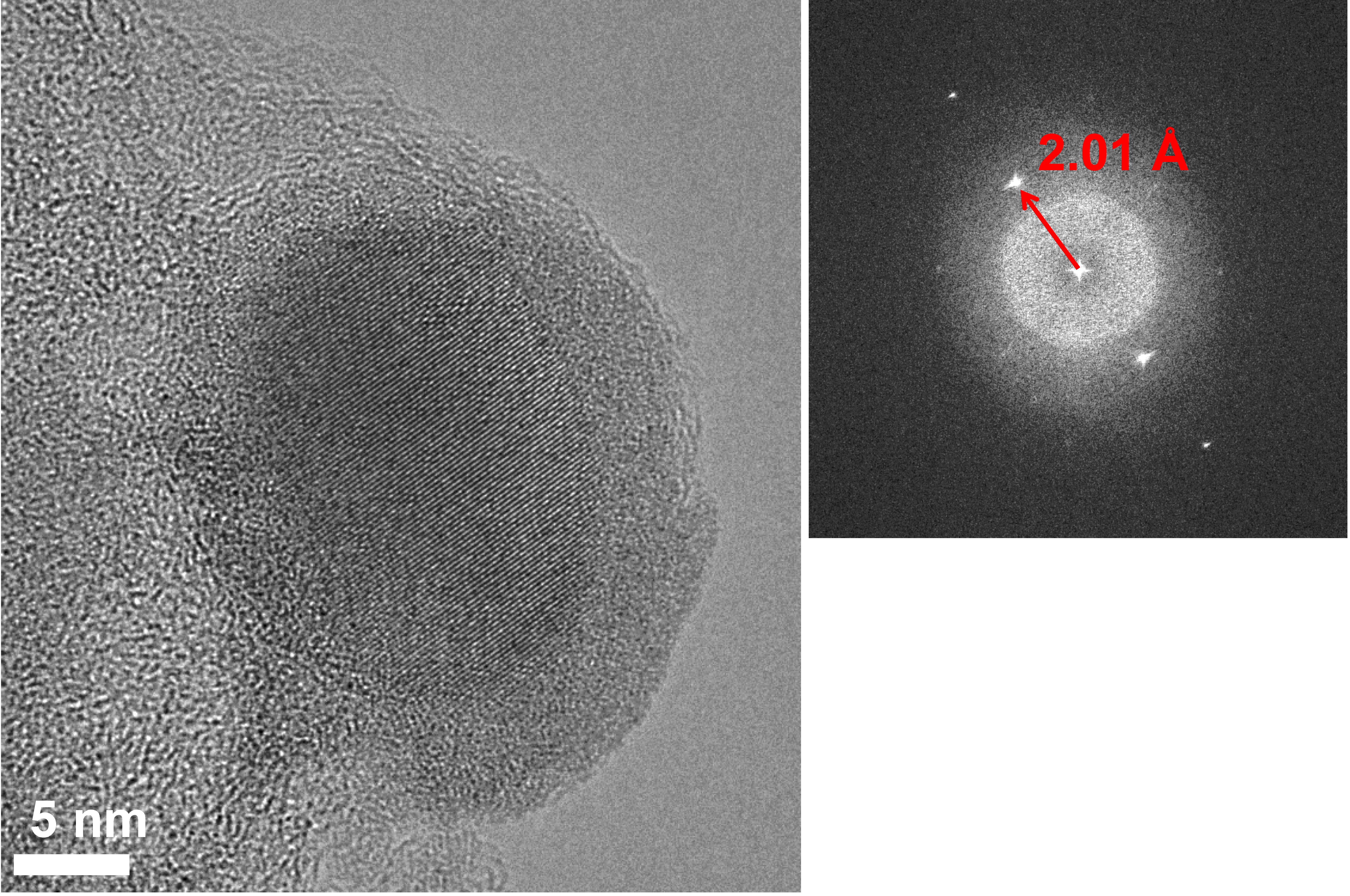


Figure S2. TEM of a tin nanoparticle surrounded by a thick amorphous carbon layer resulting from the decomposition of the polymer precursor. The 2.01 angstrom spacing corresponds to the (211) interplanar distance of tin.


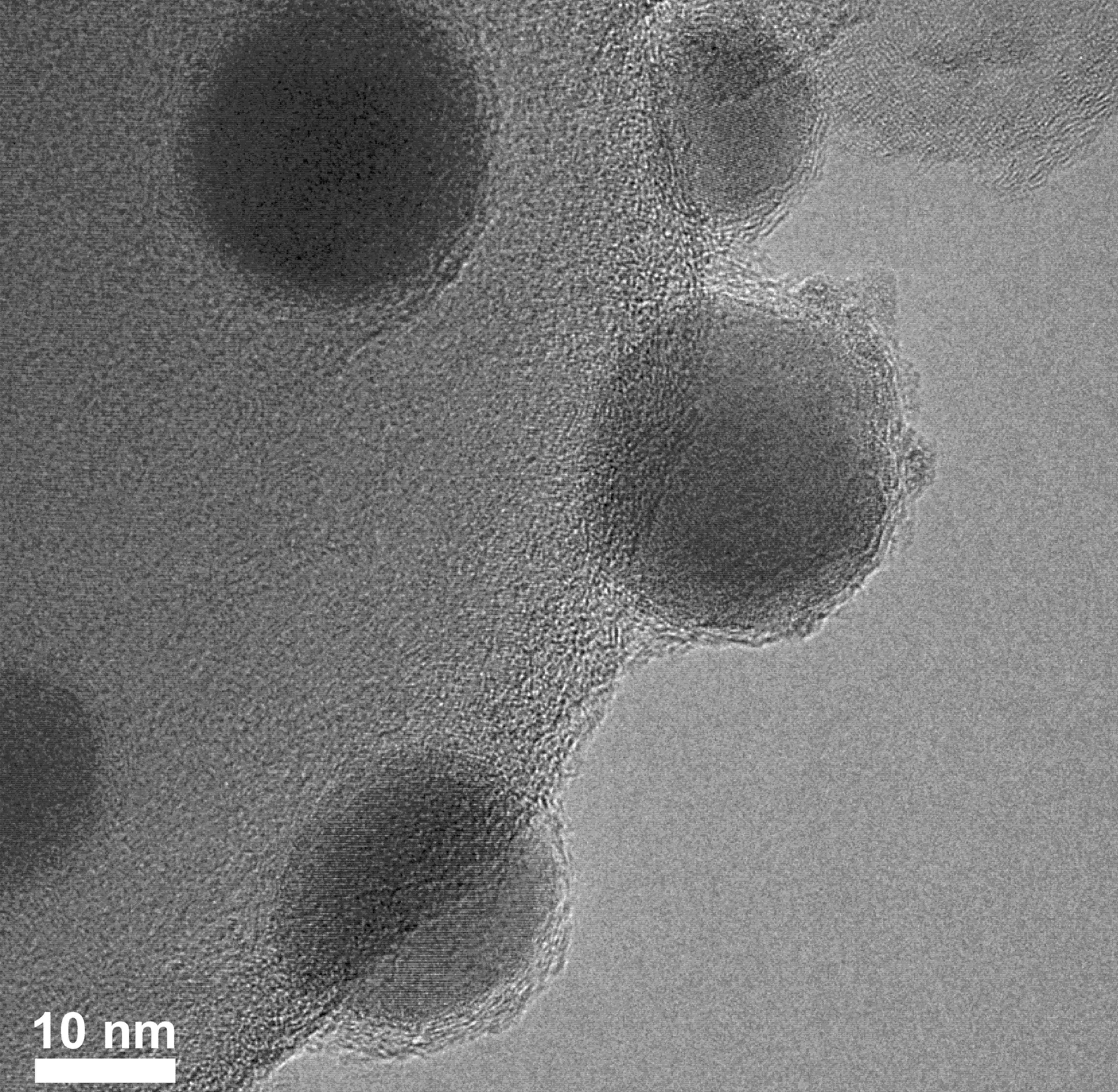


Figure S3. TEM of multiple tin nanoparticles surrounding a silicon particle. A carbon layer surrounds the overall structure.


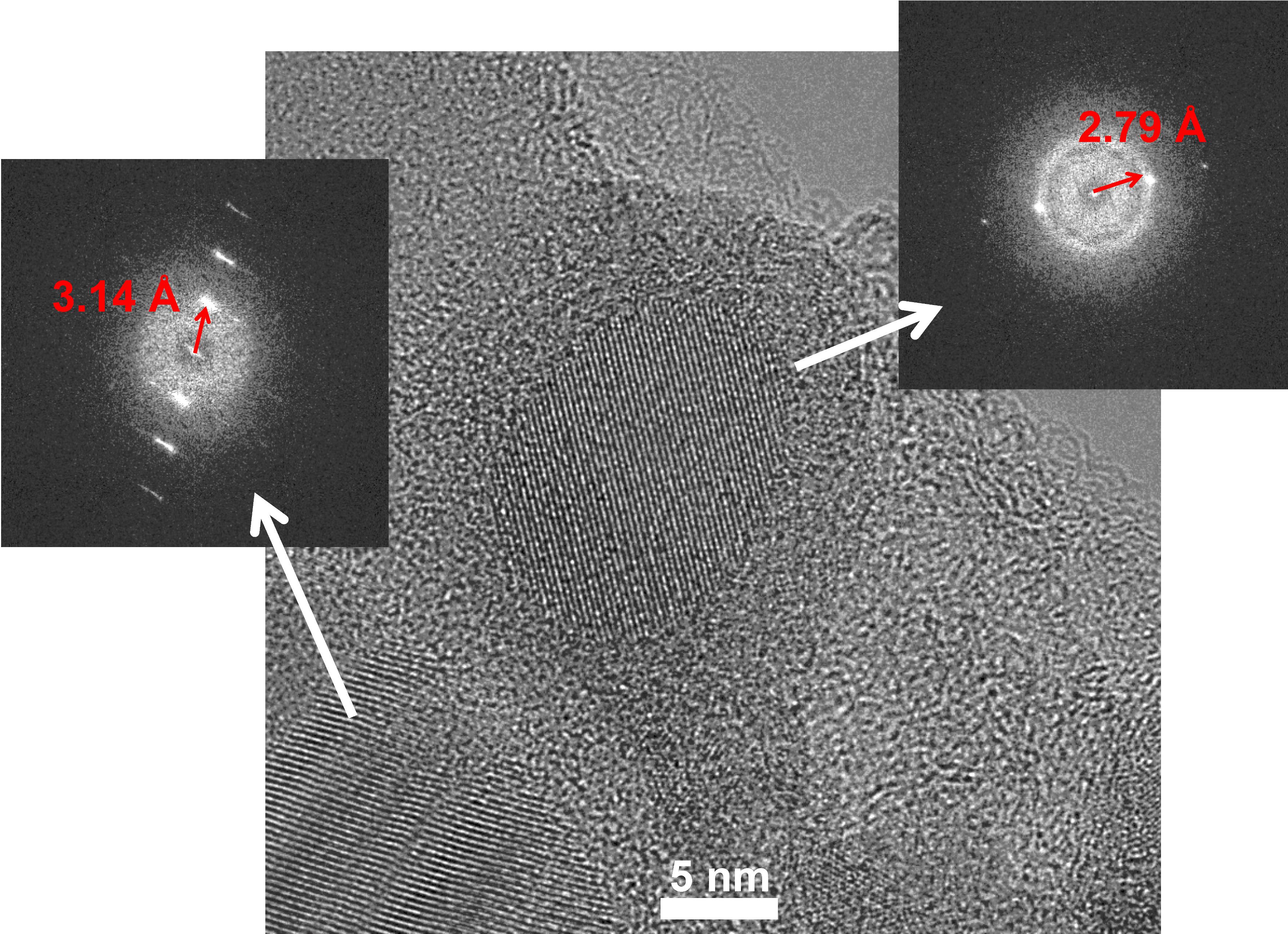


Figure S4. TEM of a tin nanocrystal (top right) anchored to a silicon nanocrystal (bottom left). A thin amorphous layer separates the two particles. The corresponding FFT patterns confirm that the bottom left particle is silicon since the 3.14 Å spacing is consistent with that between the (111) planes of silicon. The FFT pattern for the particle on the top right is consistent with the (101) spacing of tin.


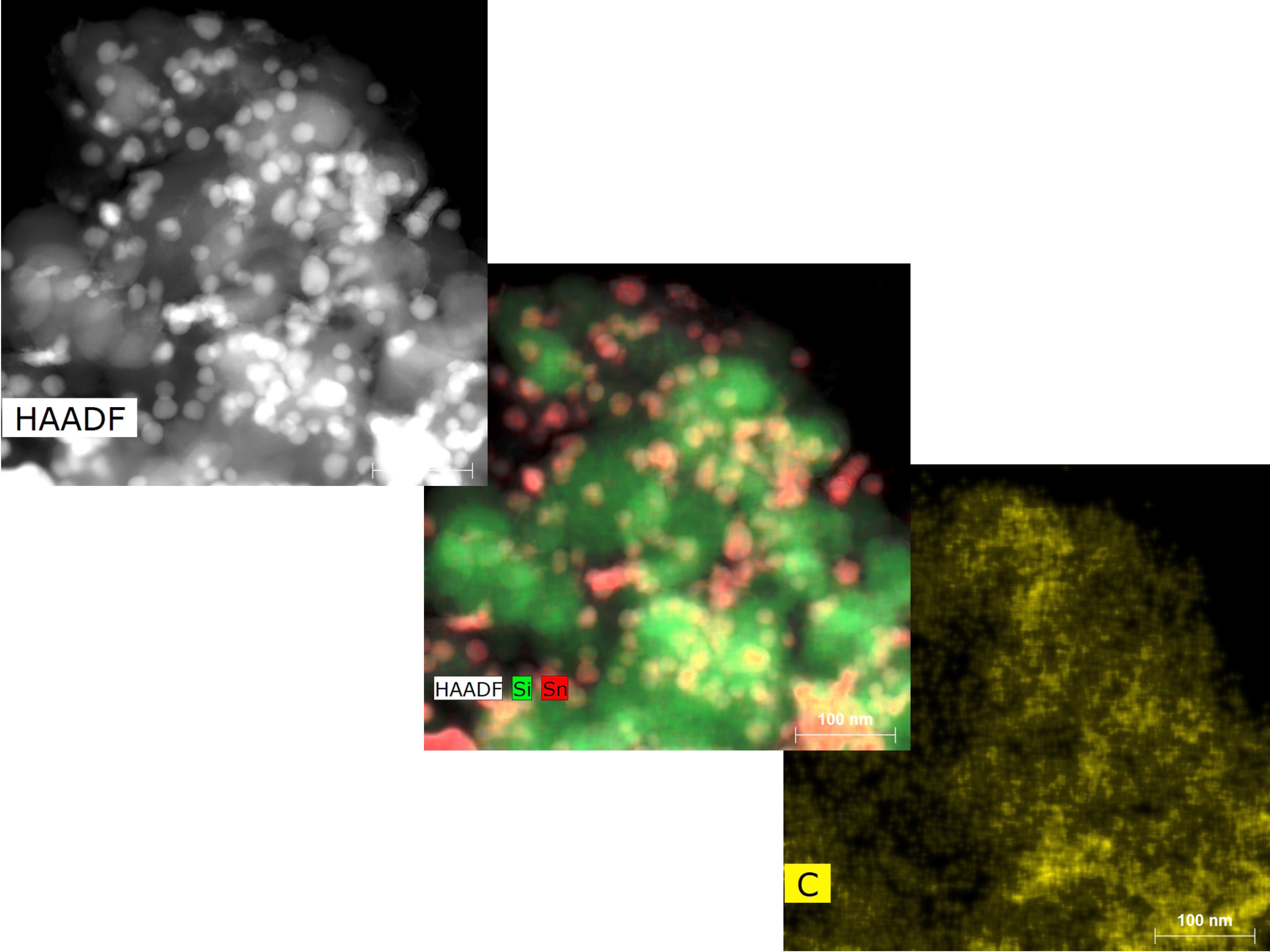


Figure S5. High-angle annular dark field image (HAADF) of the silicon-tin nanocomposite after thermal annealing, before cycling. The corresponding elemental map for silicon (green), tin (red) and carbon (yellow) are also reported to demonstrate that smaller tin nanoparticles surround the silicon particles, and a thin carbon layer coats the whole structure.

Figure S6. Cyclic voltammetry for the first three cycles of an anode containing a 9.6% weight fraction of tin.


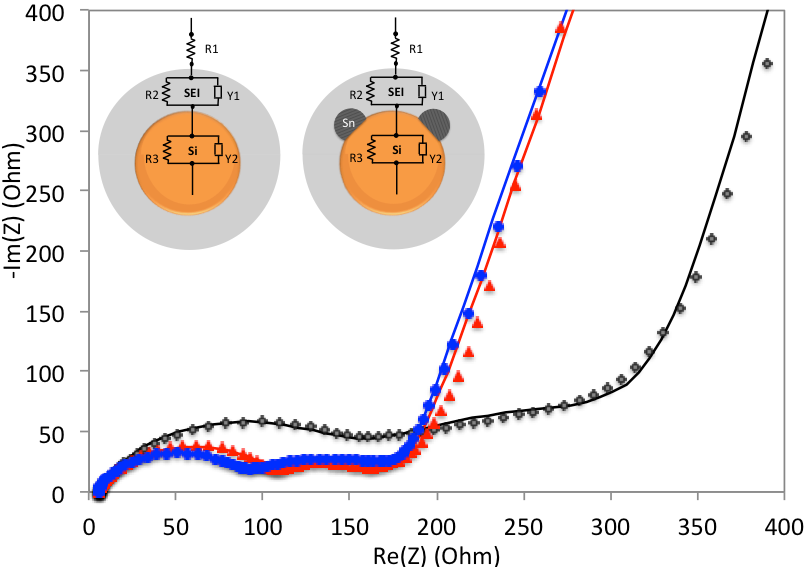


| **Electrode/R** | R1 | R2 | R3 |
| --- | --- | --- | --- |
| Without Sn | 6.2 | 140.2 | 177.8 |
| 2.2% Sn | 10.9 | 100.2 | 82.7 |
| 9.6% Sn | 5.07 | 78.09 | 93.9 |

Figure S7. EIS curve after one cycle without tin, without 2.2% (by weight) tin and with 9.6% (by weight) tin. Experimental and fit curves are shown. The table summarizes the results obtained by fitting the EIS data according to the procedure described in the manuscript.


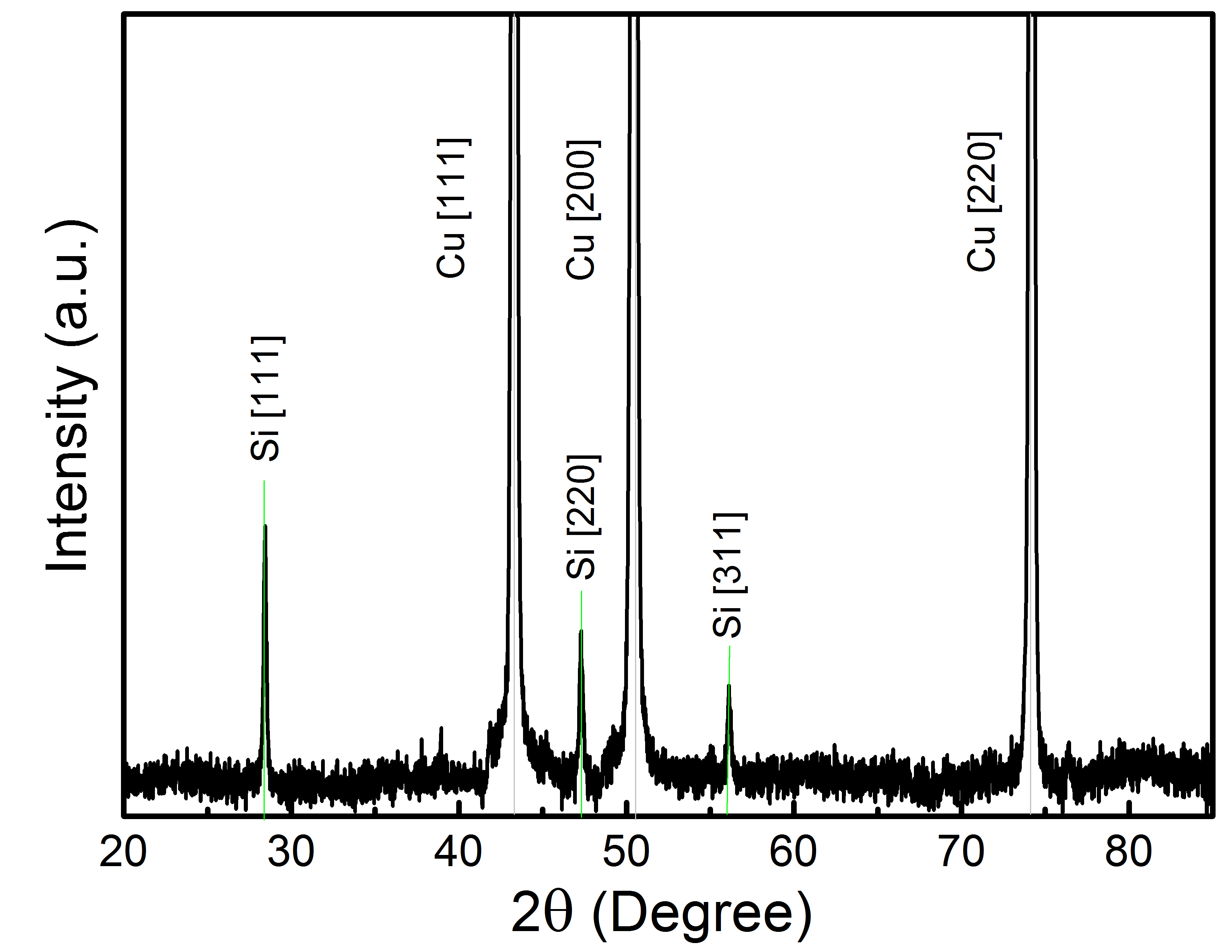


Figure S8. XRD spectrum for the sample without tin addition, after the first discharge cycle. Silicon peaks can still be detected. The copper peaks are due to the substrate.
